# Engineering ADSCs by manipulating YAP for lymphedema treatment in a mouse tail model

**DOI:** 10.3389/ebm.2024.10295

**Published:** 2024-11-20

**Authors:** Liru Hu, Nian Zhang, Chengzhi Zhao, Jian Pan

**Affiliations:** State Key Laboratory of Oral Diseases, National Center for Stomatology, National Clinical Research Center for Oral Diseases, Department of Oral and Maxillofacial Surgery, West China Hospital of Stomatology, Sichuan University, Chengdu, Sichuan, China

**Keywords:** YAP, verteporfin, ADSCs, lymphedema, lymphangiogenesis

## Abstract

Secondary lymphedema is a chronic disease associated with deformity of limbs and dysfunction; however, conventional therapies are not curative. Adipose-derived stem cells (ADSCs) based therapy is a promising way, but a single transplantation of ADSCs has limited efficacy. In this study, ADSCs were engineered *in vitro* and then transplanted into the site of lymphedema. Yes-associated protein (YAP), a crucial regulator of Hippo pathway, plays an important role in regulating stem cell functions. We examined the YAP expression in a mouse tail lymphedema model, and found that transplanted ADSCs exhibited high expression level of YAP and a large number of YAP positive cells existed in lymphedema environment. *In vitro*, the downregulation of YAP in ADSCs resulted in higher expression levels of genes related to lymphangiogenesis such as Lyve-1, VEGFR-3 and Prox-1. *In vivo*, YAP-engineered ADSCs generated abundant VEGFR-3-positive lymphatic vessels and significantly improved subcutaneous fibrosis. These results indicated that the transplantation of pre-engineered ADSCs by manipulating YAP is a promising strategy for lymphatic reconstruction.

## Impact statement

Millions of patients suffer from secondary lymphedema; however, conventional therapies are not curative. Promoting the growth of lymphatic vessels and reconstructing the lymphatic system are the key to ameliorate lymphedema. This study aimed to explore the role of Hippo pathway in regulating adipose-derived stem cell (ADSC) fate during the process of lymphangiogenesis and investigated the efficacy of engineered ADSC based therapy in lymphedema. The study showed that lymphedema-associated ADSCs exhibited high expression level of YAP and a large number of YAP positive cells existed in lymphedema environment. *In vitro*, the downregulation of YAP in ADSCs resulted in higher expression levels of genes related to lymphangiogenesis such as Lyve-1, VEGFR-3 and Prox-1. *In vivo*, YAP-engineered ADSCs generated abundant VEGFR-3-positive lymphatic vessels and significantly improved subcutaneous fibrosis. This work provided new scientific evidences for revealing the mechanism of promoting lymphangiogenesis and YAP-engineered ADSC based therapy for patients suffering from lymphedema.

## Introduction

Lymphedema, a prevalent progressive ailment, arises from impaired lymphatic vessel functionality, leading to the swelling of local tissue and subsequent discomfort and dysfunction. This condition significantly compromises the quality of life for patients [1, 2]. The goal of treatment is to alleviate symptoms, impede the advancement, and mitigate the potential hazard of lymphedema. By far, medical and surgical treatments of lymphedema are ineffective in restoring the normal functions of the lymphatic system, thereby exposing patients to the potential of experiencing recurring symptoms [3, 4]. Thus, it is urgent to find a novel method targeting in promoting reconstruction of lymphatic systems for lymphedema.

Mesenchymal stem cells (MSCs) have demonstrated great therapeutic benefits both in clinical trials and fundamental assays [5, 6]. MSCs can help tissue regeneration by secreting cytokines, chemokines, and growth factors [7]. The use of MSCs in skin wound healing and other soft tissue repairing has achieved significant progress in recent years [8, 9]. Adipose-derived stem cells (ADSCs) were first isolated and identified in 2002, and obtaining ADSCs is less invasive than other types of MSCs which makes them a good candidate for regenerative medicine [10–12]. Promoting the growth of lymphatic vessels is the key to ameliorate lymphedema. With the development of stem cell therapy, transplantation of MSCs has achieved the purpose of repairing and rebuilding the lymphatic network, thus regarded as an ideal therapy for lymphedema [13–15]. In previous studies, the use of ADSCs in the treatment of lymphedema significantly led to volume reduction and subjective improvement both in animals and humans, and no adverse reactions were reported [16–19]. Further research on the mechanism of how ADSCs promote lymphangiogenesis will help to optimize and maximize the efficacy of ADSC-assisted therapy. Therefore, we focused on the possible signaling pathway in lymphangiogenesis to find the targets for engineering ADSCs for stem cell therapy.

The Hippo pathway is crucial in regulating cell proliferation apoptosis, differentiation and development by affecting target genes through the key transcription factor TEAD, together with its coactivator Yes-associated protein (YAP) [20–23]. Hyperactivation of YAP promoted stem cell proliferation but inhibited terminal differentiation in many tissues including intestine, lung and skin [24–26]. And YAP activation promoted tissue fibrosis by regulating the activation of myofibroblasts. The inhibition of YAP expression is associated with poor fibrogenesis in livers and kidneys, conversely, fibroblasts with YAP overexpression caused accumulation of extracellular matrix components and lung fibrosis [27, 28]. Fibrosis, an irreversible pathological change, is one of the most important pathological characteristics in the process of lymphedema. Therefore, improving fibrosis is of great significance in alleviating lymphedema [1, 2]. Thus, we speculated that manipulating the expression of YAP in ADSCs facilitated cell differentiation and improved fibrosis, helping make a recovery from lymphedema.

Based on the above results, it can be speculated that the downregulation of YAP expression could promote the differentiation of ADSCs toward lymphatic endothelial cells (LECs). This study aimed to investigate the efficacy of manipulating YAP in ADSCs based therapy in a mouse lymphedema model, so as to provide new ideas for the clinical treatment of lymphedema.

## Materials and methods

### Cell culture

White adipose tissue in the inguinal was collected from postnatal 7-day C57BL/6 mice (purchased from Dashuo Experimental Animal Limited Company, Chengdu, China). Harvested tissue was digested in collagenase I (C2-BIOC, Sigma, St. Louis, MO, United States) for 1 h and centrifuged at 1,500 rpm for 5 min to obtain the cell-debris pellet. The pellet was resuspended in αMEM medium (12561056, Gibco, Grand Island, NY, United States) supplemented with 10% FBS (A5669701, Gibco) and 1% penicillin/streptomycin (15140122, Gibco) in an incubator. The medium was changed every other day. Cells were passaged when reaching 80% confluence and the third passages of ADSCs were used in follow-up experiments. Then 1 × 10^6^ ADSCs were seeded in each well of the 6-well plate. For the lymphatic transdifferentiation of ADSCs, VEGFC (100 ng/mL, HY-P77864, MedChem Express, Monmouth Junction, NJ, United States) was used for 7 days. For YAP engineering, verteporfin (20μM, HY-B0146, MedChem Express) was used for 48 h.

### Animal model establishment

All procedures were registered and approved by the Ethics Committees of the State Key Laboratory of Oral Diseases, West China School of Stomatology, Sichuan University (WCHSIRB-D-2022-277). Six-week-old female adult C57BL/6 mice weighing an average of 20 g (purchased from Dashuo Experimental Animal Limited Company, Chengdu, China) were used in this study. All animals were maintained with free access to laboratory food and water.

To establish the lymphedema tail model, the mice were anesthetized by inhalation of 5% isoflurane. Throughout the surgical interventions, anesthesia was sustained with 2% isoflurane to ensure the animals remained unconscious. A 2-mm wide full-thickness circumferential skin piece was dissected at 20 mm distal from the tail base, removing superficial lymphatic vessels in the process. Following the subcutaneous injection of 0.1% Evans blue dye into the tip of the tail, the deep lymphatic vessels were cut carefully without damaging accompanied veins. To maintain a moist and infection-free environment, the surgical site was covered with sterile gauze and treated with erythromycin ointment for 24 h.

### Isolation of lymphedema-associated ADSCs

CM-Dil dye stock was prepared as recommended by the manufacturer (V22888, Thermo Fisher Scientific, Waltham, MA, United States) to label ADSCs for 5 min at 37°C, and then for an additional 15 min at 4°C. Next, the cell suspension was centrifuged and then the cells were washed twice in sterile PBS. Subsequently, local subcutaneous transplantation of 2 × 10^6^ CM-Dil-labeled ADSCs along with 30 μL PBS was performed at lymphedema site in the lymphedema tail 1 week after surgery. Then mice were euthanized after 48 h, and soft tissue of the entire tail was digested to obtain single-cell suspension for flow cytometric cell sorting of CM-Dil-labeled ADSCs.

### Quantitative real-time PCR

At the designated times, cells were collected to test the relative mRNA expression of LEC-related markers and TAP. The reverse transcription of the total RNA was completed by using the HiScript II Q RT SuperMix for qPCR kit (Vazyme Biotech, China). The synthesized cDNA templates were used to do quantitative real-time PCR by using SYBR Green PCR reagents (Bio-Rad, United States). The ΔΔCt (the threshold cycle) values were calculated and the results were expressed as the ratio of the mRNA copies of target genes to that of GAPDH gene (reference gene). The primers involved in our study were showed as follows: 5′- CAG CAC ACT AGC CTG GTG TTA -3′ (forward) and 5′- CGC CCA TGA TTC TGC ATG TAG A-3′ (reverse) for Lyve-1; 5′- ACA GAA GCT AGG CCC TAC TG -3′ (forward) and 5′-ACC CAC ATC GAG TCC TTC CT -3′ (reverse) for VEGFR-3; 5′- AGA AGG GTT GAC ATT GGA GTG A-3′ (forward) and 5′- TGC GTG TTG CAC CAC AGA ATA -3′ (reverse) for Prox-1; 5′- TGT TTA TGG GAC AGT CCG GG -3′ (forward) and 5′- CGA GGA CGG ATT CAT CTT TCT GG -3′ (reverse) for YAP; 5′- TGG ATT TGG ACG CAT TGG TC -3′ (forward) and 5′- TTT GCA CTG GTA CGT GTT GAT -3′ (reverse) for GAPDH.

### Immunofluorescence staining

After removing the medium, the cells were washed with PBS three times before being fixed for half an hour with 4% paraformaldehyde. Subsequently, the cells were permeabilized using 0.5% Triton X-100 (T8200, Solarbio, Beijing, China) and blocked for 30 min at room temperature using 2% FBS. Following an overnight incubating at 4°C with primary antibodies, the cells were then treated for 1 h at room temperature with secondary antibodies and 10 min at room temperature with 4′,6-diamidino-2-phenylindole dihydrochloride (DAPI, C0065, Solarbio). An Olympus inverted fluorescence microscope was used to capture the images. Paraffin tissue sections were deparaffinized in xylene and rehydrated through graded ethanol solutions. After being repaired by EDTA (C1034, Solarbio), the sections were blocked by 5% BSA for 30 min at room temperature and incubated with primary antibodies overnight at 4°C. Following steps were the same as cellular immunofluorescence described above. A confocal microscope (N-STORM & A1, Nikon) was used to capture the images.

The primary and secondary antibodies used in this study were as follows: anti-VEGFR-3 (1: 200 dilution, Hunan Biotechnology), anti-YAP (1: 200 dilution, Proteintech), goat anti-rabbit 488 (1:200 dilution, Hunan Biotechnology).

### Western blot

At the designated times, nuclear and cytosolic extracts from ADSCs were prepared with a Nuclear Protein Extraction Kit (P0027, Beyotime, Shanghai, China). And total proteins were collected by using RIPA (V900854, Sigma) for ADSC lysis. Subsequently, the proteins run on 10% gels (PG112, Epizyme Biotech, Shanghai, China) and were transferred to poly- (vinylidene fluoride) (PVDF) membranes. Following an overnight incubating at 4°C with primary antibodies, the membranes were treated for 1 h at room temperature with secondary antibodies. Chemiluminescence images were taken by a chemiluminescence machine (Bio-Rad, Hercules, CA, United States). The primary antibodies used in this study were as follows: anti-GAPDH (1: 5,000 dilution, ET1601-4, Hunan Biotechnology, Hangzhou, China), anti-H3 (1: 5,000 dilution, M1309-1, Hunan Biotechnology), anti-VEGFR-3 (1: 1,000 dilution, ER65750, Hunan Biotechnology) and anti-YAP (1: 2,000 dilution, 13584-1-AP, Proteintech).

### Tube formation assay

The 24-well plate was coated with Matrigel (356234, Corning Incorporated, Corning, NY, United States), then the gel was allowed to polymerize at 37°C for 30 min. The ADSCs were seeded on the gel (1 × 10^5^ cells/well) and images were taken 12 h after seeding.

### The transplantation of ADSCs

After surgery, the mice of tail lymphedema model were randomly divided into three groups to and received weekly local subcutaneous injections around the incision gap.

The control group was administered 30 μL PBS. The ADSC group was administered 2 × 10^6^ ADSCs along with 30 μL PBS [29–31]. The ADSC-verteporfin group received the same amount of Yap-engineered ADSCs along with 30 μL PBS.

### Quantitative evaluation of lymphedema

The degree of lymphedema was assessed by the circumference of the tail at two specific sites (5 mm and 10 mm distal from the incision) weekly. The measurements were conducted three times, and the averages were computed.

### Histological and immunohistochemical staining

The lymphedema tails were obtained at the second and fourth weeks after surgery. The tissues were preserved in a 4% paraformaldehyde solution for 48 h and then placed in paraffin following decalcification. The samples were divided into sections at intervals of 5 μm, starting from a distance of 10 mm away from the surgical site. To evaluate the degree of subcutaneous fibrosis, the paraffin-embedded tissue sections were stained with Masson’s trichrome stain following conventional methods. And Picro-Sirius red staining was performed to analyze the collagen type I and III by using the commercial Kit (MM1036, Maokang bio, Shanghai, China) according to the manufacturer’s instructions.

For immunohistochemical staining, after being repaired by EDTA (C1034, Solarbio), the slides were rinsed with water and incubated with the primary antibodies overnight at 4°C. Then the slides were rinsed and incubated with the corresponding secondary antibody for 30 min followed by 3,3′-diaminobenzidine and hematoxylin staining, respectively. The primary antibodies used in this study were as follows: anti-YAP (1: 50 dilution, ET1611-69, Hunan Biotechnology) and anti-VEGFR-3 (1: 200 dilution, ER65750, Hunan Biotechnology).

### Statistical analysis

On the basis of the assessment of the normal distribution of the data, all experiments were performed at least in triplicate. Data were presented as mean ± SD. When parametric test assumptions were met, the statistical significance was determined by one-way analysis of variance (ANOVA using the SPSS 21.0 software). Statistical significance was set at *p* < 0.05.

## Results

### YAP expression increased in lymphedema-associated ADSCs

**FIGURE 1:**
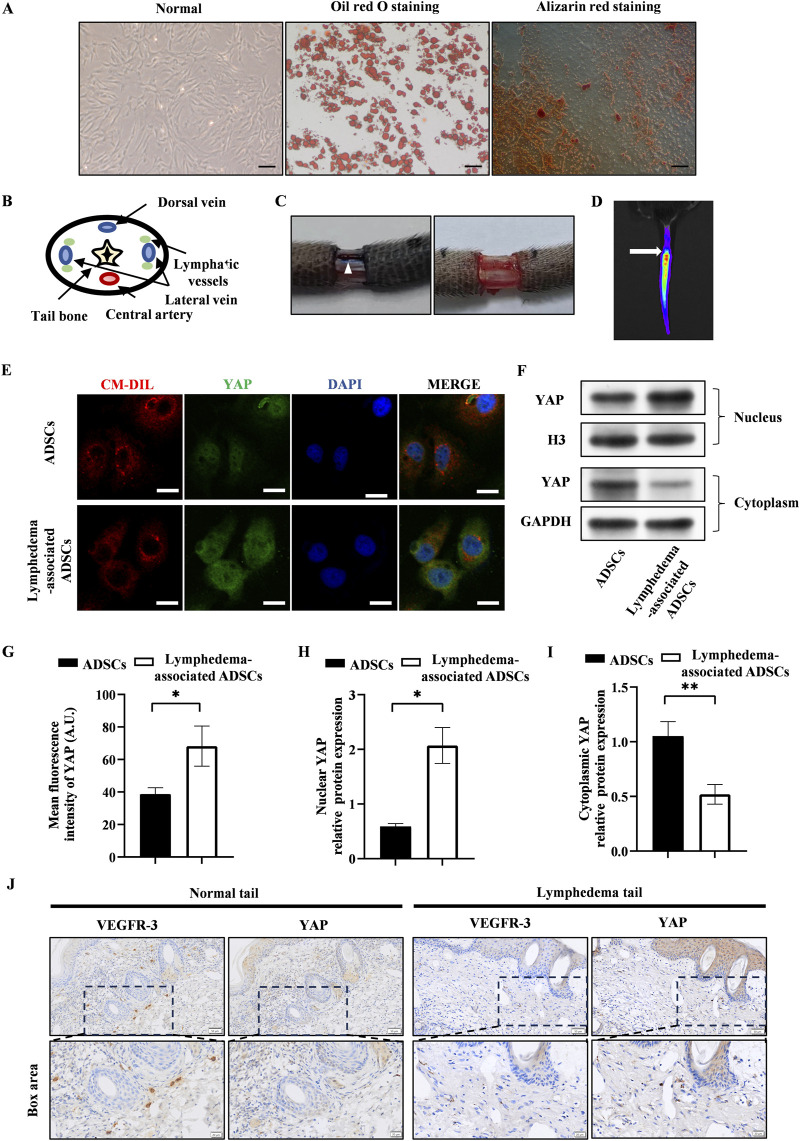
YAP expression increased in lymphedema-associated ADSCs. **(A)** Passage 3 ADSCs exhibited the characteristic spindle-like shape. Oil red staining and alizarin red staining showed the osteogenic and adipogenic potential of ADSCs, respectively. Scale bar = 200 μm. **(B)** The anatomical diagram of tail cross section. **(C)** Representative images of the surgical site before and after removing the lymphatic vessels during surgery, and the white triangle indicates the lymphatic vessels. **(D)** Representative image of *in vivo* fluorescence observed in tail transplanted with CM-Dil labeled ADSCs (the arrow points to the surgical incision). **(E)** Representative micrographs of YAP immunostaining in lymphedema-associated ADSCs compared with ADSCs from normal adipose tissue. Scale bar = 20 µm. **(F, H, I)** Western blot showed the increased nuclear YAP expression and decreased cytosolic YAP expression in lymphedema-associated ADSCs compared with ADSCs from normal adipose tissue. **(G)** Immunofluorescence intensity analysis of YAP. **(J)** The immunohistochemical staining of the lymphedema tail and normal tail. Cells in lymphedema tail had low expression level of VEGFR-3 and high expression level of YAP. Scale bar = 20 μm and 50 µm. Bars: means ± standard deviation. n = 3 in each group, *P < 0.05, **P < 0.01.

The passage 3 ADSCs exhibited the characteristic spindle-like shape, and their ability to differentiate into adipogenic and osteogenic lineages was validated by using oil red staining and alizarin red staining (Figure 1A). The lymphedema tail model was established by the surgical removal of the lymphatic vessels that accompanied the lateral veins (Figures 1B, C). After the transplantation of CM-Dil-labeled ADSCs, the presence of ADSCs were tracked by the IVIS Spectrum *in vivo* optical imaging system within the tail (Figure 1D).

To assess the effect of lymphedema environment on YAP expression in ADSCs, we isolated ADSCs from the lymphedema tail to evaluate the YAP expression in lymphedema-associated ADSCs. Immunofluorescence staining detected significantly higher expression of YAP in lymphedema-associated ADSCs compared to normal ADSCs (Figures 1E, G). As YAP activity is regulated by its nuclear translocation, western blot was used to investigate the nuclear and cytosolic expression of YAP. The results of western blot showed that the nuclear expression of YAP increased in lymphedema-associated ADSCs while the cytosolic expression decreased compared to normal ADSCs (Figures 1F, H, I). Meanwhile, we evaluated YAP expression in the lymphedema tail by immunohistochemical staining. Cells in the lymphedema tail had low expression level of VEGFR-3 and high expression level of YAP compared to the normal tail (Figure 1J). Based on these data, we proposed that YAP expression was positively correlated with the development of lymphedema.

### YAP expression decreased after the lymphatic endothelial transdifferentiation of ADSCs

**FIGURE 2:**
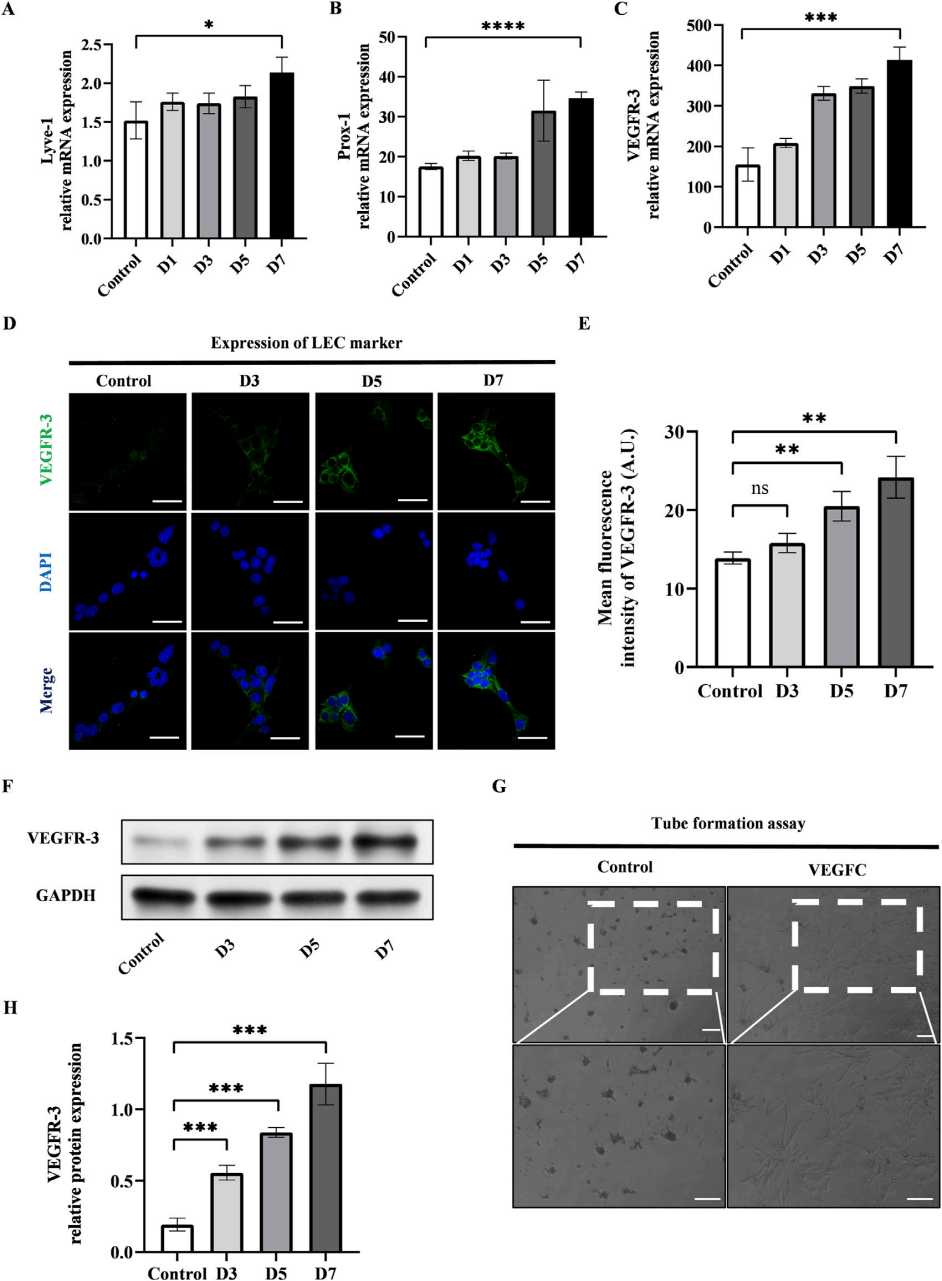
VEGFC successfully induced lymphatic endothelial transdifferentiation of ADSCs. **(A–C)** PCR tests showed the upregulation of VEGFR-3, Prox-1, Lyve-1 during the lymphatic endothelial transdifferentiation of ADSCs. **(D)** VEGFR-3 as a typical LEC marker was detected by immunofluorescence staining after VEGFC-induction at the indicated times. Scale bar = 50 μm. **(E)** Immunofluorescence intensity analysis of VEGFR-3. **(F, H)** Western blot showed the increased VEGFR-3 expression during the lymphatic endothelial transdifferentiation of ADSCs. **(G)** ADSCs were seeded on Matrigel after 7-day induction, and tube formation was evaluated at 12 h postseeding. VEGFC group generated tube-like structure while control group did not exhibit tubes. Scale bar = 100 μm. Bars: means ± standard deviation. n = 3 in each group, ns: no significant, **P < 0.01, ***P < 0.001.

We conducted the lymphatic endothelial transdifferentiation of ADSCs by using VEGFC at a concentration of 100 ng/mL. We observed the upregulation of LEC-related markers including Lyve-1, Prox-1, and VEGFR-3 at mRNA level after 7-day induction (Figures 2A–C). The confirmation of this was further supported by the immunofluorescence staining and western blot of VEGFR-3, as seen in Figures 2D–F, H. Additionally, the tube formation assay was conducted after 7-day induction of VEGFC to measure the ability of ADSCs to form tubes and promote the growth of LECs. This evaluation is considered the gold standard for determining the impact of VEGFC on lymphangiogenesis. Figure 2G showed that the VEGFC group exhibited tubulogenesis while the control group did not demonstrate the formation of tube-like structures.

**FIGURE 3:**
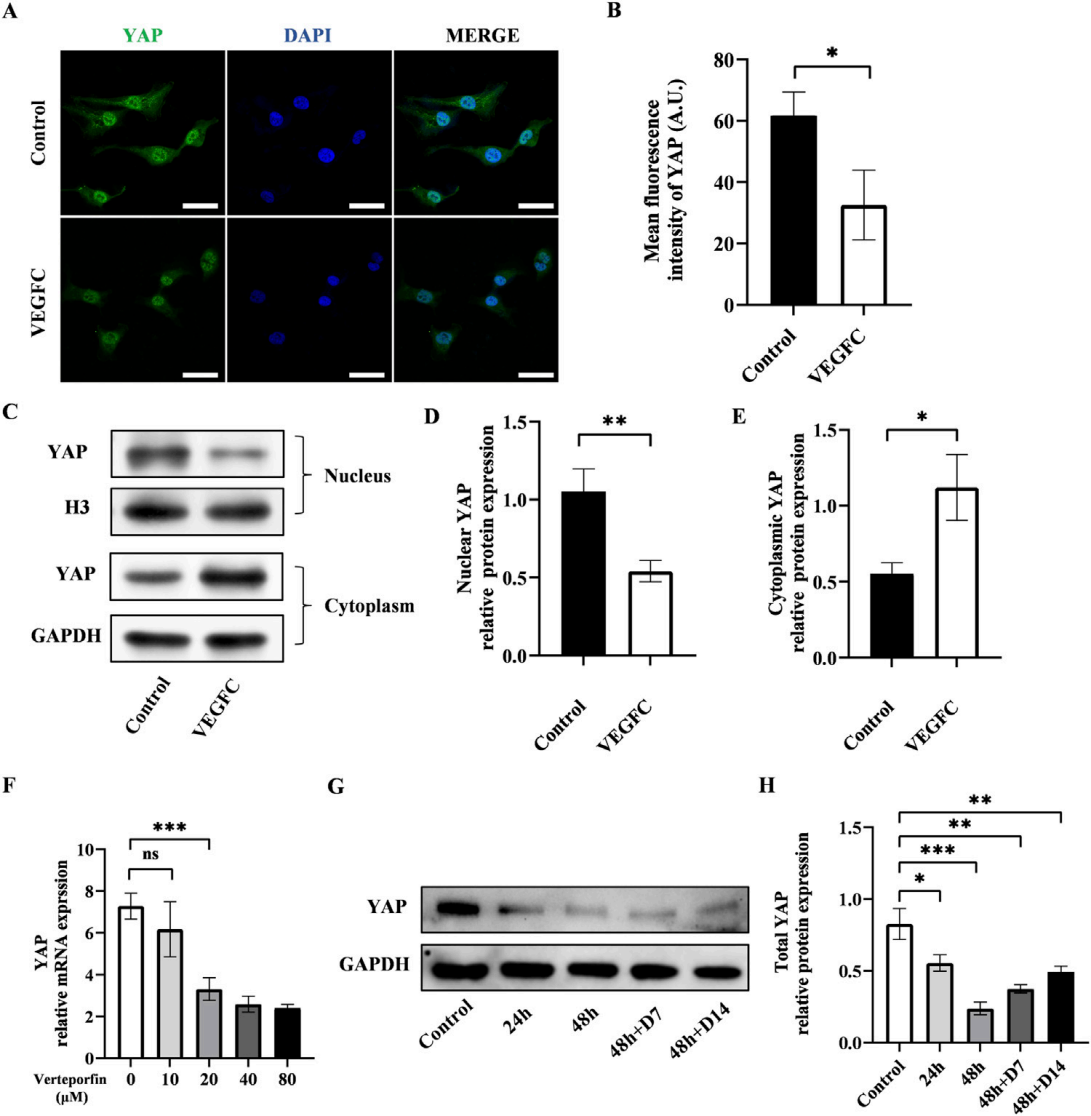
Effect of lymphatic endothelial transdifferentiation and verteporfin on the expression of YAP. **(A)** Immunostaining of YAP in the control group and VEGFC group. Scale bar = 50 μm. **(B)** Immunofluorescence intensity analysis of YAP. **(C–E)** Western blot showed the decreased nuclear YAP expression and increased cytosolic YAP expression in ADSCs after lymphatic endothelial transdifferentiation. **(F)** PCR test showed that verteporfin suppressed the expression of YAP in ADSCs at the concentration of 20 μM. **(G**, **H)** Western blot showed the continuously inhibitory effect of verteporfin on YAP expression in ADSCs. Bars: means ± standard deviation. n = 3 in each group; ns, no significant, *P < 0.05, **P < 0.01, ***P < 0.001.

Next, we investigated the alteration of YAP after the lymphatic endothelial transdifferentiation. To do this, we conducted immunofluorescence staining to evaluate the YAP expression and found that the expression of YAP exhibited a significant drop in the VEGFC group compared to the control group (Figures 3A, B). The results of western blot showed lower nuclear YAP expression and higher cytosolic expression in the VEGFC group compared to the control group (Figures 3C–E). Based on these data, we speculated that there was a decrease in YAP phosphorylation, leading to a reduction in its nuclear translocation.

The changes in YAP phosphorylation status might be associated with the transdifferentiation process of ADSCs and could potentially influence lymphangiogenesis.

### The downregulation of YAP enhanced the lymphatic endothelial transdifferentiation of ADSCs

Verteporfin as a widely recognized YAP inhibitor, was employed to manipulate YAP expression in this investigation. The results of PCR showed that verteporfin suppressed the YAP expression in ADSCs at the concentration of 20 μM (Figure 3F). Meanwhile, we performed western blot to determine the duration of inhibitory effect on YAP after a 48-hour treatment of 20 μM verteporfin (Figures 3G, H). The results showed that the YAP expression was significantly lower in the 48h + D7 group and 48h + D14 group compared to the control group. The inhibitory effect of verteporfin on YAP could last for at least 2 weeks after the 48-hour treatment of verteporfin.

**FIGURE 4:**
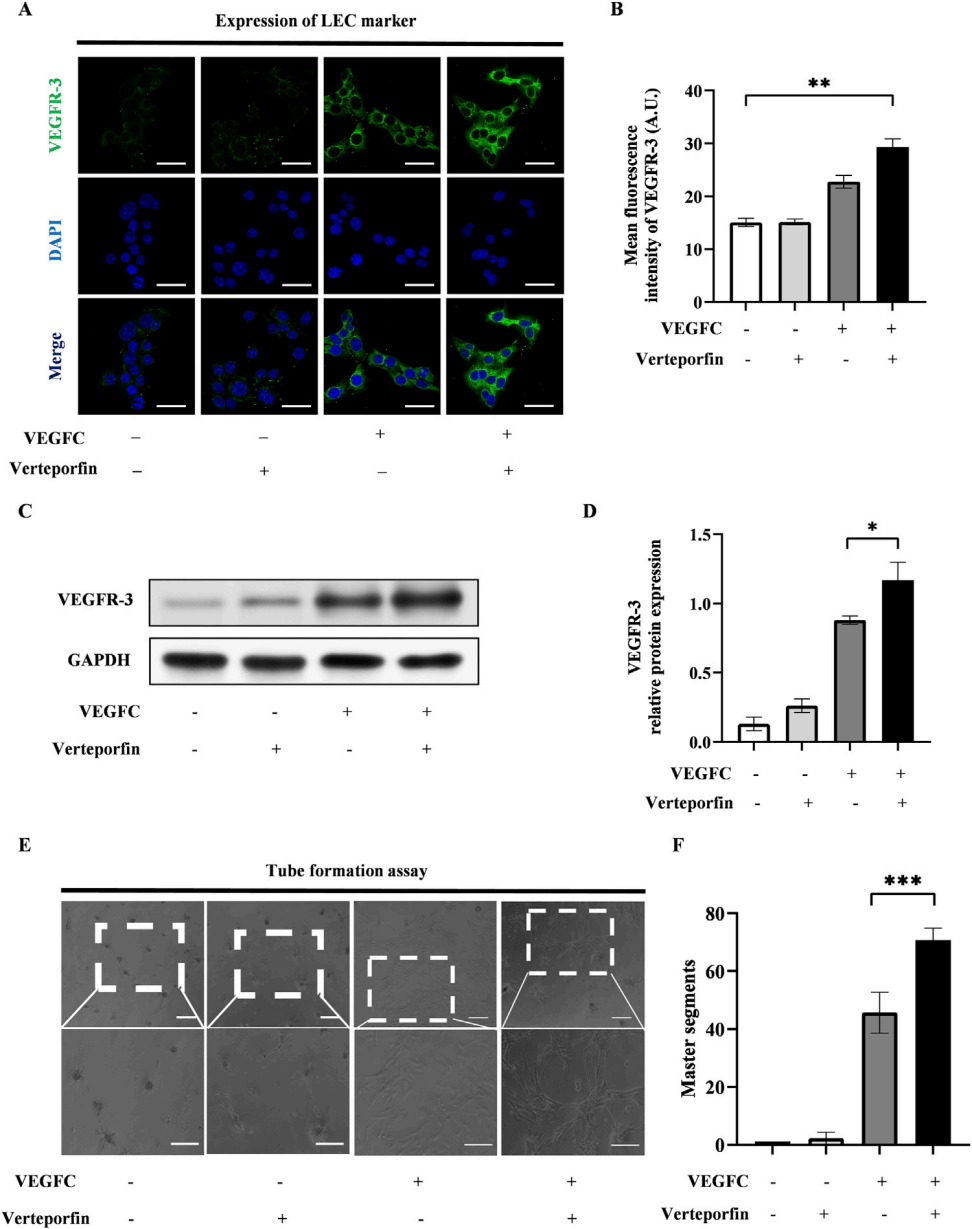
The downregulation of YAP enhanced the lymphatic endothelial transdifferentiation of ADSCs *in vitro*. 20 μM verteporfin preconditioning for 48 h downregulated the expression of YAP in ADSCs. Under this inhibitory effect, higher expression levels of VEGFR-3 can be detected after differentiation of ADSCs, and larger density of tube formation can be observed. **(A)** Lymphatic endothelial transdifferentiation of ADSCs was conducted after 20 μM-verteporfin preconditioning for 48 h, and immunofluorescence staining indicated higher level of VEGFR-3. Scale bar = 50 μm. **(B)** Immunofluorescence intensity analysis of VEGFR-3. **(C, D)** Higher expression level of VEGFR-3 was confirmed by western blot. **(E, F)** The tube formation assay showed that VEGFC (+) verteporfin (+) group generated more tube-like structure than VEGFC (+) verteporfin (−) group. And quantification of master segments was analysed. Scale bar = 200 μm. Bars: means ± standard deviation. n = 3 in each group, ns: no significant, *P < 0.05, **P < 0.01, ***P < 0.001.

To investigate the effect of YAP downregulation on the lymphatic endothelial transdifferentiation, ADSCs were induced by VEGFC following a 48-hour pretreatment of verteporfin. The expression level of VEGFR-3 increased significantly in the VEGFC (+) verteporfin (+) group compared to the VEGFC (+) verteporfin (−) group, which was improved by immunofluorescence staining (Figures 4A, B) and western blot (Figures 4C, D). In the tube formation assay, the VEGFC (+) verteporfin (+) group generated a greater number of tubes compared to the VEGFC (+) verteporfin (−) group. Interestingly, tubes were observed in the VEGFC (−) verteporfin (+) group (Figures 4E, F). These data indicated that YAP downregulation could enhance the lymphatic endothelium differentiation of ADSCs.

### Changes in mouse tail edema after surgery

**FIGURE 5:**
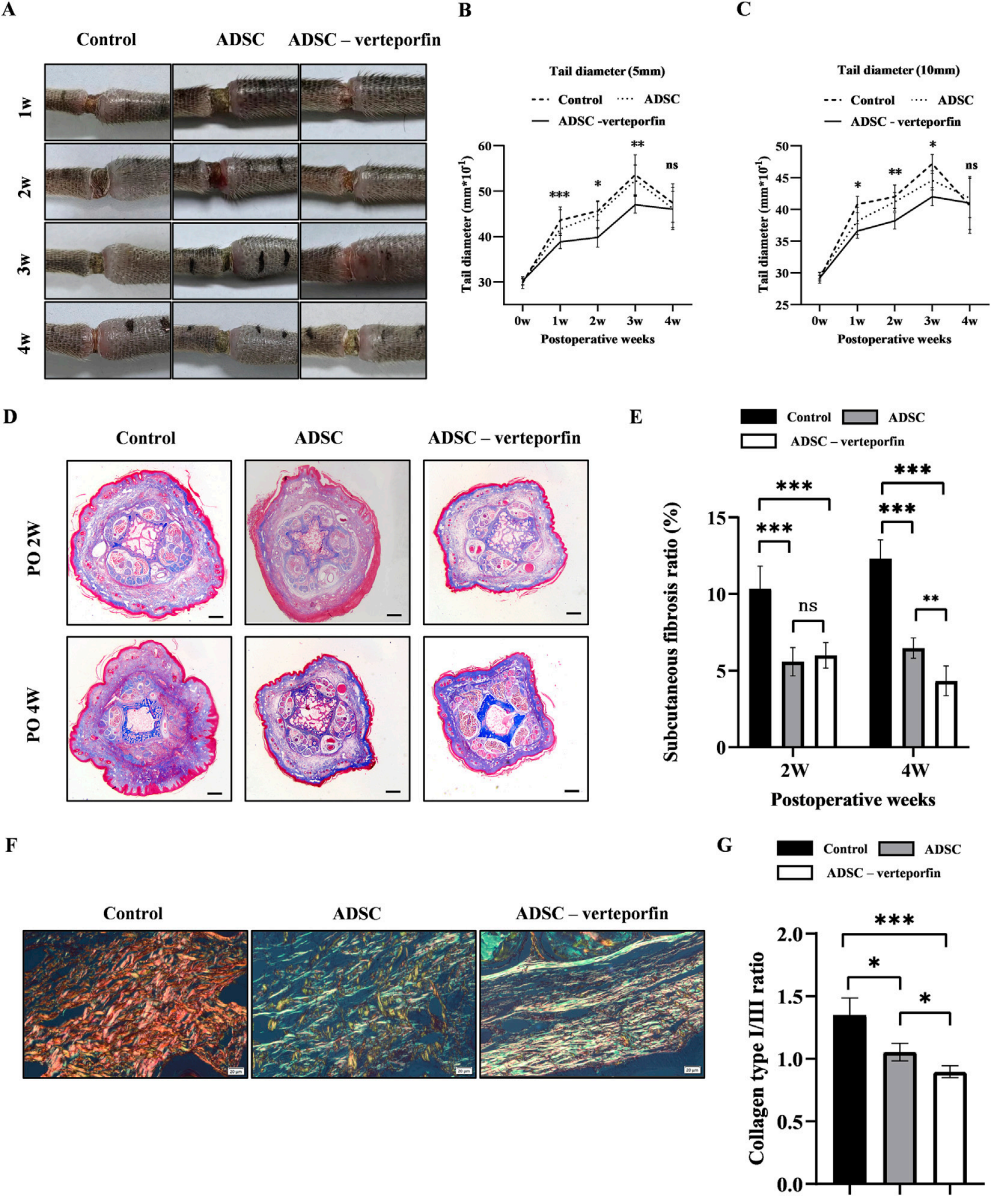
YAP-downregulation ADSCs reduced the degree of swelling and improved fibrosis mouse tail lymphedema models. **(A)** Representative images of the mouse tail at 1, 2, 3, and 4 weeks after surgery. **(B, C)** Tail diameter was measured before and after surgery at the site of 5 mm and 10 mm distal from the incision. **(D)** Masson staining of the subcutaneous tissue of mouse tail at 2 and 4 weeks after surgery. Scale bar = 500 μm. **(E)** Statistical analysis of subcutaneous fibrosis ratio. **(F)** Representative results for Picro-Sirius red staining of mouse tail at 2 and 4 weeks after surgery. Red-yellow fibers represented collagen type I and green fibers with weak birefringence represented collagen type III. Scale bar = 20 μm. **(G)** Collagen type I/III ratio showed that more Collagen type III was produced in ADSCs (VEGFC+ verteporfin) group. n = 5 in each group, ns: no significant, *P < 0.05, **P < 0.01, ***P < 0.001.

Following the surgical removal of the lymphatic vessels, a noticeable accumulation of fluid was noted in the mouse tail caused by the obstruction of lymphatic drainage. Figure 5A displayed representative photographs of the mouse tail at 1, 2, 3, and 4 weeks after surgery.

The diameter of the mouse tail gradually increased over time after surgery, reaching a peak at 3 weeks (Figures 5B, C). The tail diameter of the ADSC -verteporfin group increased the least compared to the other groups. The distinction became more apparent when considering the physiological growth of the tail during the experimental period. These data suggested that local transplantation of YAP-downregulation ADSCs could alleviate lymphedema.

### YAP-downregulation ADSCs reduced fibrosis in lymphedema

Fibrosis is one of the important pathological characteristics in the process of lymphedema.

Therefore, Masson staining was conducted to visualize the presence of collagen in the subcutaneous tissue at 2 and 4 weeks after surgery. An accumulation of densely packed collagen fibers, stained blue, was observed in the subcutaneous tissue while muscle fibers were stained red (Figure 5D). The control group had higher subcutaneous fibrosis ratio at 2 and 4 weeks after surgery compared to the ADSC group and ADSC-verteporfin group (Figure 5E). It demonstrated that the local transplantation of ADSCs could reduce the degree of fibrosis in lymphedema. And the ADSC-verteporfin group had lower subcutaneous fibrosis ratio at 4 weeks compared to the ADSC group which indicated a more pronounced benefit of YAP-downregulation ADSCs in improving fibrosis. In Picro-Sirius red staining, red-yellow fibers represented collagen type I and green fibers with weak birefringence represented collagen type III (Figure 5F). At 4 weeks, significantly higher collagen type I/III ratio was found in the control group compared to the ADSC group and ADSC-verteporfin group. And the ADSC-verteporfin group showed the lowest collagen type I/III ratio (Figure 5G).

### YAP-downregulation ADSCs promoted lymphangiogenesis during lymphedema development

**FIGURE 6:**
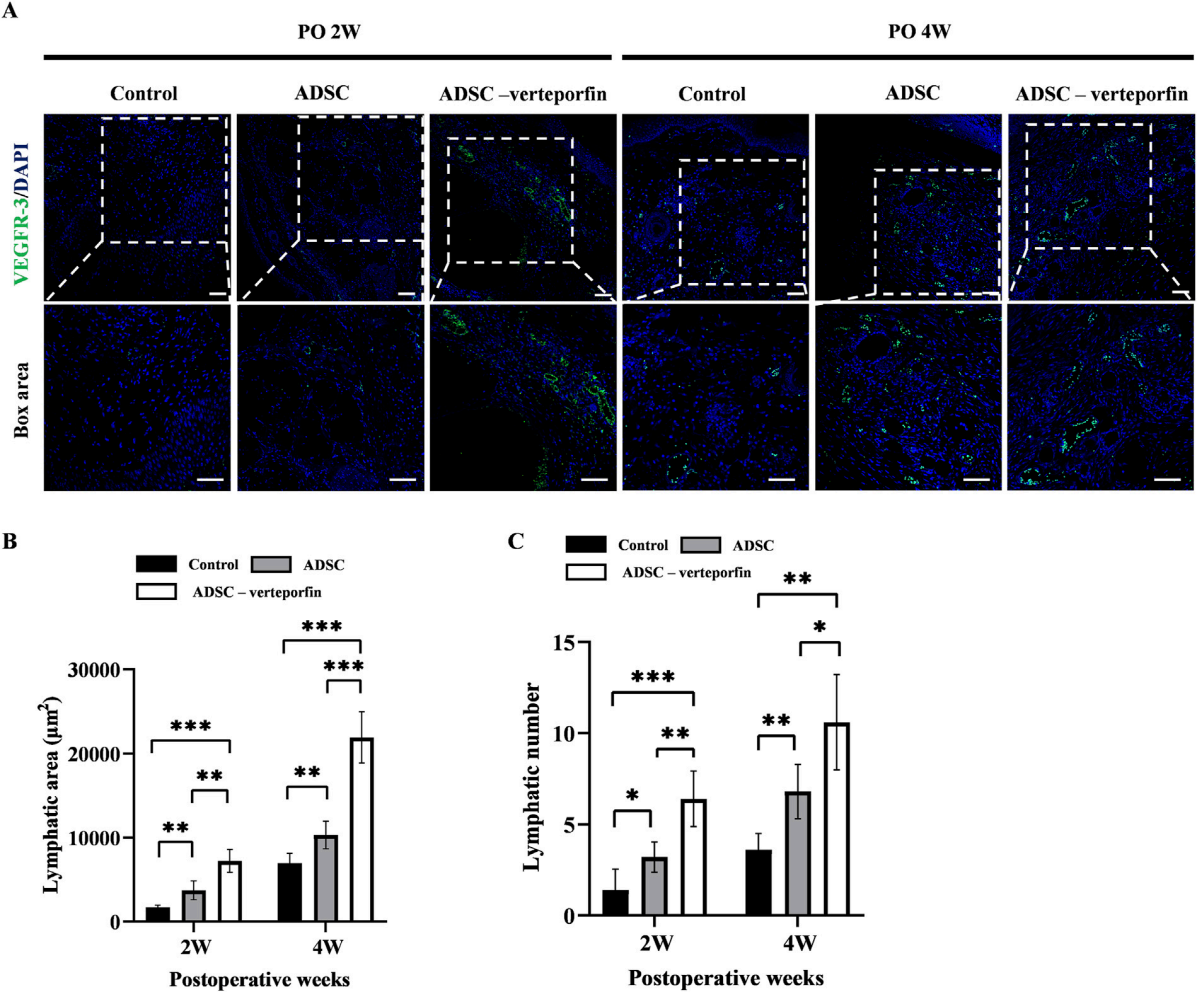
The regeneration of lymphatic vessels in the mouse tail lymphedema model. **(A)** Subcutaneous VEGFR-3^+^ lymphatic vessels were detected by immunofluorescence staining. Scale bar = 200 μm. **(B)** Statistical analysis of the area of lymphatic vessels. **(C)** Statistical analysis of the number of lymphatic vessels. n = 5 in each group, *P < 0.05, **P < 0.01, ***P < 0.001.

In order to assess the formation of lymphatic vessels, we performed immunofluorescence staining for VEGFR-3 on the mouse tails at 2 and 4 weeks after surgery (Figure 6A). By calculating the area and number of lymphatic vessels, we can quantitatively analyze the lymphangiogenesis in each group (Figures 6B, C). The results showed that the control group had significantly smaller lymphatic area and number compared to at 2 and 4 weeks after surgery compared to the ADSC group and ADSC-verteporfin group. It demonstrated that the local transplantation of ADSCs could promote lymphangiogenesis. And the ADSC-verteporfin group exhibited significantly bigger lymphatic area and number at 2 and 4 weeks after surgery compared to the ADSC group which indicated a more pronounced benefit of YAP-downregulation ADSCs in lymphangiogenesis.

## Discussion

Stem cells based lymphedema therapies have attracted attention in recent years for its great regeneration ability in reducing edema which has been proved by clinical and basal studies [32]. The pathology of lymphedema is the destruction of lymphatic vessels and excessive fibrosis. Stem cells can promote the growth of lymphatic vessels to reconstruct local lymphatic system at the same time. They have therapeutic effects in anti‐inflammatory and anti-fibrosis, which makes stem cells ideal for lymphedema treatment [33]. However, a major concern of stem cell therapy is that the transplanted stem cell population is of highly heterogeneous which means the therapeutic efficacy of ADSC transplantation may vary among studies.

The crosstalk between MSCs and LECs is crucial for lymphangiogenesis [34]. The key of MSCs therapy lies in secreting growth factors, and VEFGC is the most important factor among them. Activation of VEGFR-3 by VEGFC results in the phosphorylation of protein kinase B and extracellular regulatory kinase, which can enhance the migration, proliferation, and survival of LECs [35]. Several research examining the preclinical animal model of acquired lymphedema provide evidence for therapeutic lymphangiogenesis through the activation of VEGFC/VEGFR-3 signaling pathways [36–41]. For this study, we used VEGFR-3 as the main marker associated with LECs, and we found that lymphangiogenesis was positively correlated with high expression of VEGFR-3 both *in vitro* and *in vivo*. Then increased growth factors derived from MSCs promoted sprouting of lymphatics from LECs by binding to their high affinity receptors. Therefore, engineered MSCs with enhanced secretion function are potentially be utilized towards an effective lymphedema treatment.

YAP expresses abundantly in diverse stem cell populations *in vivo* and *in vitro*, and participates in various physiological and pathological processes by driving stem cell behavior and regeneration [42]. Abnormal expression of YAP drives the development of aging and tumorigenesis associated with stem cell dysfunction which can be reversed by YAP targeted therapies [43]. At the onset of stem cell differentiation, YAP activity is depressed and YAP gene silencing caused the loss of stemness [44]. Altogether, YAP can be regarded as a regulator in balancing progenitor and differentiated cells in different physiological and pathological environment.

In this study, we found that YAP was upregulated in transplanted ADSCs and a large number of YAP positive cells in lymphedema site were seen. These results suggested that YAP played an important role in lymphedema by affecting ADSCs. Verteporfin is an authorized pharmaceutical for the treatment of macular degeneration. Currently, it is employed as a small molecule inhibitor for YAP-TEAD and has demonstrated anti-cancer properties in several forms of solid tumors [45–47]. We used verteporfin to manipulate YAP expression of ADSCs, and the upregulation of VEGFR-3 and increased tube forming capacity indicated that suppressing YAP could induce the lymphogenic phenotype of ADSCs. Furthermore, the transplantation of ADSCs preconditioned with verteporfin exhibited reducing swelling and better lymphangiogenesis in the early stages of secondary lymphedema in in vivo experiments. These results indicated that the downregulation of YAP in ADSCs played an important role in promoting lymphangiogenesis. Engineered ADSCs based on manipulating YAP expression is a practical way to reconstruct lymphatic circulation.

The development of lymphedema is commonly attributed to a feedback loop that involves local inflammation, lymphatic fibrosis, and the deposition of adipose tissue [48]. Chronic inflammation-induced fibrosis plays a key role in the pathophysiology of this disease, which decreases collecting lymphatic pumping, and impairs collateral lymphatic formation. Numerous studies clearly showed the association between fibrosis and lymphedema [49], similarly, in our study, severe fibrosis occurred in untreated lymphedema, and the verteporfin-ADSC therapy showed promise in mitigating the extent of subcutaneous fibrosis. Collagen type I and III are important fiber components in determining the tensile strength of soft tissue. Collagen type I increases stiffness, whereas collagen type III increases the flexibility of tissues [50]. The collagen type I/III ratio is regarded as an informative marker in biological processes and pathological conditions. Altered collagen type I/III ratio was reported to be associated with abnormal regeneration pattern in scar development [51]. However, collagen type I/III ratio is poorly investigated in lymphedema. In this study, we found that verteporfin-ADSC therapy decreased the collagen type I/III ratio in treating lymphangiogenesis. The regulation of collagen type I and III as well as the collagen type I/III ratio may serve as a new target for further investigation. In addition to ability of promoting lymphangiogenesis, engineered ADSCs played a positive role in suppressing fibrosis to improve the outcomes of lymphedema.

## Data Availability

The datasets presented in this study can be found in online repositories. The names of the repository/repositories and accession number(s) can be found in the article/supplementary material.
